# Phenocopy of a heterozygous carrier of X-linked retinitis pigmentosa due to mosaicism for a *RHO* variant

**DOI:** 10.1038/s41598-020-80400-3

**Published:** 2021-01-08

**Authors:** Ine Strubbe, Caroline Van Cauwenbergh, Julie De Zaeytijd, Sarah De Jaegere, Marieke De Bruyne, Toon Rosseel, Stijn Van de Sompele, Elfride De Baere, Bart P. Leroy

**Affiliations:** 1 Department of Ophthalmology, Ghent University Hospital, Ghent, Belgium; 2 Department of Head & Skin, Ghent University, Ghent, Belgium; 3 Center for Medical Genetics, Ghent University Hospital, Ghent, Belgium; 4 Department of Biomolecular Medicine, Ghent University, Ghent, Belgium; 5 Division of Ophthalmology, Children’s Hospital of Philadelphia, Philadelphia, PA USA; 6 Center for Cellular and Molecular Therapeutics, Children’s Hospital of Philadelphia, Philadelphia, PA USA

**Keywords:** Genetics research, Clinical genetics, Gene expression, Medical genetics

## Abstract

We describe both phenotype and pathogenesis in two male siblings with typical retinitis pigmentosa (RP) and the potentially X-linked RP (XLRP) carrier phenotype in their mother. Two affected sons, two unaffected daughters, and their mother underwent detailed ophthalmological assessments including Goldmann perimetry, color vision testing, multimodal imaging and ISCEV-standard electroretinography. Genetic testing consisted of targeted next-generation sequencing (NGS) of known XLRP genes and whole exome sequencing (WES) of known inherited retinal disease genes (RetNet-WES). Variant validation and segregation analysis were performed by Sanger sequencing. The mutational load of the *RHO* variant in the mother was assessed in DNA from leucocytes, buccal cells and hair follicles using targeted NGS. Both affected sons showed signs of classical RP, while the mother displayed patches of hyperautofluorescence on blue light autofluorescence imaging and regional, intraretinal, spicular pigmentation, reminiscent of a carrier phenotype of XLRP. XLRP testing was negative. RetNet-WES testing revealed *RHO* variant c.404G > C p.(Arg135Pro) in a mosaic state (21% of the reads) in the mother and in a heterozygous state in both sons. Targeted NGQSS of the *RHO* variant in different maternal tissues showed a mutation load between 25.06% and 41.72%. We report for the first time that somatic mosaicism of *RHO* variant c.404G > C p.(Arg135Pro) mimics the phenotype of a female carrier of XLRP, in combination with heterozygosity for the variant in the two affected sons.

## Introduction

Retinitis Pigmentosa (RP) is a group of progressive inherited retinal diseases (IRDs) characterized by rod-degeneration preceding cone-degeneration. RP has a worldwide prevalence of 1:4000, making it a leading cause of visual disability^1^. In keeping with the differential involvement of the photoreceptor systems, patients with RP usually first display symptoms of night blindness, followed by progressive loss of vision in the midperipheral visual fields. In the later stages of the disease, the cone-rich center is affected as well, with concomitant decline of visual acuity. The age of onset varies greatly^1–3^.

RP can be inherited in an autosomal dominant (ADRP, 15–25% of cases), autosomal recessive (ARRP, 5–20%), or X-linked fashion (XLRP, 5–15%). The remainder are mostly simplex cases (30–55%)^4,5^. ADRP patients generally show a milder phenotype, with longer preservation of central vision. The recessive and X-linked forms tend to be more severe in progression. A special entity are the female carriers of X-linked RP. Talib et al*.* recently compared a cohort of 125 heterozygous carriers of X-linked *retinitis pigmentosa GTPase regulator* (*RPGR*) mutations and described a wide spectrum of phenotypes, ranging from no symptoms to a severe phenotype. Overall, 23% of heterozygous carriers displayed a complete phenotype, although usually milder than in affected hemizygous males^6^. Mutations in *RPGR* account for most cases of XLRP, while mutations in the *RP2* and *OFD1* genes are less frequent^7^.

Here, we report the clinical and genetic characterization of the first case of a mosaic carrier of a dominant *RHO* c.404G > C p.(Arg135Pro) variant, showing clinical signs suggestive for XLRP, and her two sons, heterozygous for the variant, with typical signs of RP.

## Material and methods

**Figure 1:**
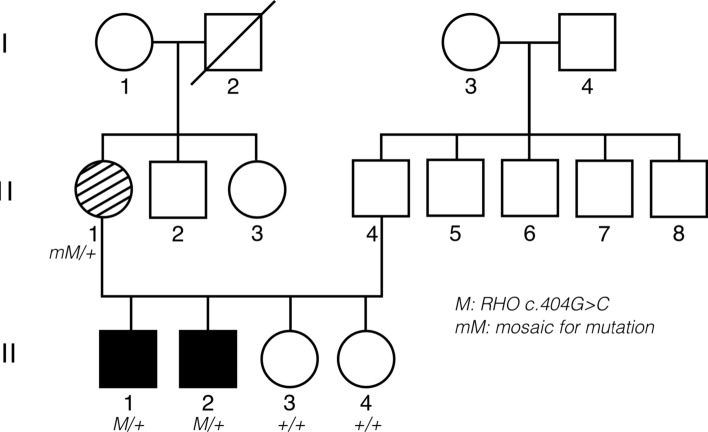
Pedigree of the family and segregation analysis of the *RHO* variant. Filled symbol: affected individual. Striped symbol: mildly affected female, mimicking carriership of XLRP. M/ + : heterozygous *RHO* variant c.404G > C p.(Arg135Pro). + / + : homozygous wild type. mM/ + : mosaicism for c.404G > C p.(Arg135Pro).

The proband (III.1), his siblings (III.2, III.3, III.4) and his mother (II.1) underwent a full clinical and genetic work-up (Fig. 1).

This study was conducted following the tenets of the Declaration of Helsinki and ethical approval was given by the ethics committee at Ghent University Hospital/Ghent University. All patients were enrolled in a clinical context. Verbal and written informed consent for study participation was obtained from all patients.

### Clinical study

A detailed review of the medical records was performed and symptoms were registered. Best-corrected visual acuity measurements, slit-lamp biomicroscopy, fundoscopy, Goldmann perimetry with visual field areas determined with the V4e, I4e and I2e targets and color vision testing with Ishihara, Hardy-Rand-Rittler pseudo-isochromatic plates and the Farnsworth Panel D-15 arrangement test were conducted. Multimodal imaging included spectral-domain optical coherence tomography (OCT) (Heidelberg Spectralis, Heidelberg Engineering, Heidelberg, Germany), white light fundus pictures (Topcon Medical Systems, Tokyo, Japan) and fundus autofluorescence-imaging (FAF) using both short wavelength (blue) and near-infrared light (Heidelberg HRA2, Heidelberg Engineering, Heidelberg, Germany). Full-field flash electroretinograms were recorded according to the standards as described by the International Society for Clinical Electrophysiology of Vision standard (ISCEV)^8^.

### Genetic and genomic study

Peripheral blood samples were collected in EDTA tubes and DNA was extracted using the QiAmp, Gentra Puregene Cell kit (Qiagen, Antwerp, Belgium) or the ReliaPrep Large-Volume HT gDNA Isolation System (Promega, Leiden, the Netherlands) according to the manufacturer’s protocol. DNA from buccal mucosa and hair follicles was extracted using NaOH and Tris-HCl. The coding and exon–intron regions of the *RPGR* (ENST00000378505), *RP2* (ENST00000218340) genes, as well as the deep intronic *OFD1* IVS9 + 706A > G mutation were tested in the proband (III.1) using targeted next-generation sequencing (NGS). PCR-enrichment of all coding exons and flanking intron sequences was followed by sequencing by synthesis (MiSeq, Illumina). A variant of class 3, 4 or 5 was confirmed by Sanger sequencing. Individuals II.1 and III.1 underwent whole exome sequencing on a HiSeq 3000 (Illumina) after exome enrichment using the SureSelect Human All Exon V6 kit (Agilent). Read alignment and variant calling were performed using the CLC Genomics Workbench (v7.5.4). Variants were annotated and filtered using in-house software. Variants were scored heterozygous or homozygous in case of a variant allele frequency of 20–69% and minimum 70% respectively. A selection of 265 RetNet genes was assessed (gene panel version v5). Variants were assessed on the basis of predictions done in Alamut HT/Alamut Batch. (Likely) pathogenic variants were confirmed using Sanger sequencing. Nucleotide numbering was done following HGVS-guidelines (http://www.hgvs.org) with nucleotide ‘A’ of the ATG as ‘c.1’. Classification of variants was based on the ACMG guidelines with adaptations^9–12^. Targeted testing of the *RHO* variant (II.1, III.1, III.2, III.3, III.4) was performed using Sanger sequencing of exon 2 (Ref Seq, ENST00000296271). An estimation of the mutation load in II.1 was performed on DNA extracted from blood using bidirectional Sanger sequencing of *RHO* exon 2 and data analysis using SeqPilot (JSI Medical Systems GmbH, Germany). To assess the mutation load in multiple tissues (blood, buccal mucosa and hair follicles), targeted deep sequencing was performed using a flexible targeted next-generation protocol, consisting of singleplex-PCR, followed bij NexteraXT library preparation and sequencing on a MiSeq instrument as previously described^13^. Variants were filtered with a minimum allele frequency threshold of 1%. The sequencing depth varied from 147 to 17,523 reads (after removal of duplicate reads and overlapping paired-end reads).

## Results

### Phenotypic characteristics

#### Individual III.1

**Figure 2:**
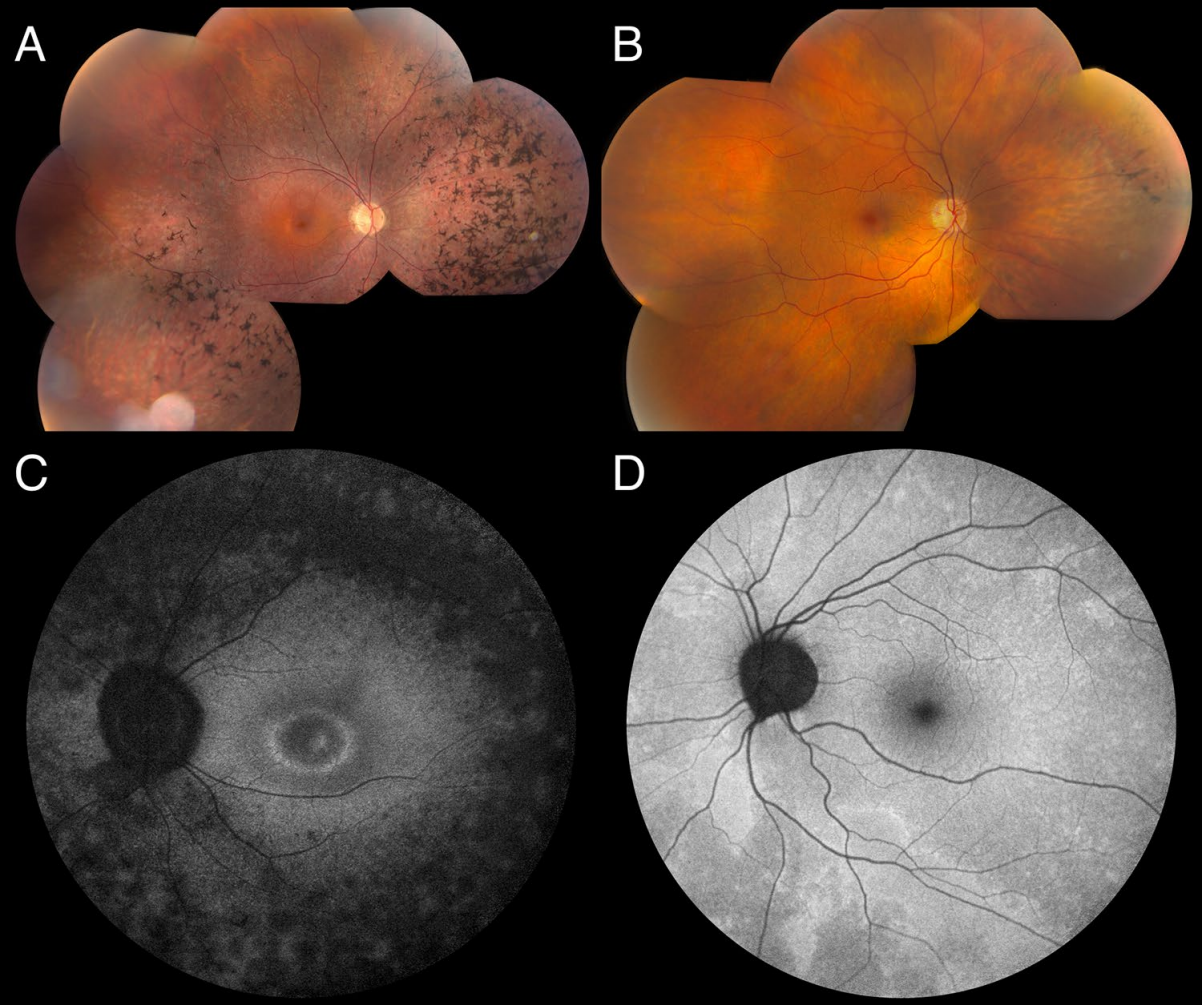
Fundus imaging. (**A**) Fundus pictures of both eyes of individual III.1 (age 28): note RPE alterations in retinal midperiphery and periphery. Spicular intraretinal pigment migrations in the periphery. (**B**) Fundus pictures of both eyes of individual II.1 (age 52): Midperipheral RPE alterations and pigment migrations, more pronounced in, but not exclusive to nasal midperiphery. (**C**) Blue light autofluorescent imaging of both eyes of individual III.1 (age 28): mottled hypo-autofluorescent periphery, with small hyper-autofluorescent ring around the macula, with a second hyperfluorescent ring around temporal vascular arcades. (**D**) Blue light autofluorescent imaging of both eyes of individual II.1 (age 52): note mosaic pattern of hyperfluorescent areas, some around the vasculature.

**Figure 3:**
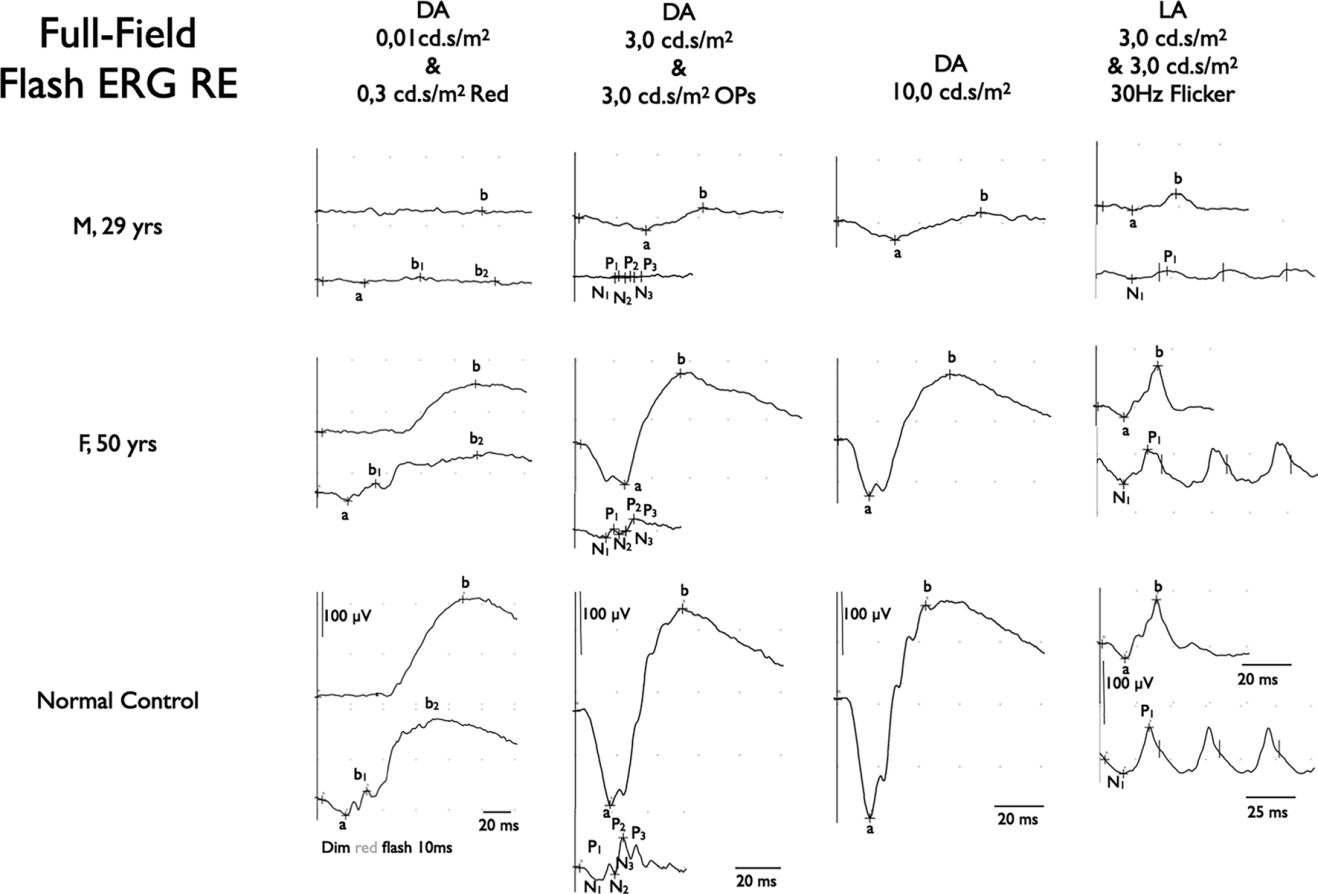
ISCEV standard full-field flash electroretinogram (ERG). (Top traces) Right eye (RE) of proband (III.1) at age 29. Scotopic ERG: complete absence of rod specific response to a dim white flash (0.01 cd.s/m2); residual response to slightly higher intensity red flash (0,3 cd.s/m2); responses to high intensity flashes (3.0 cd.s/m2 and 10.0 cd.s/m2) show significant reduction of amplitudes and major delay in peak times. Photopic ERG: response to a transient, high-intensity flash (3.0 cd.s/m2) also shows significant reduction in amplitudes and a delay of responses. This is confirmed by response to a 30 Hz high intensity (3.0 cd.s/m2) flicker stimulus. (Middle traces) RE of the mother of the proband (II.1) at age 50. Scotopic rod specific ERG: reduction of amplitude of rod specific response to a dim white flash (0.01 cd.s/m2) to about two thirds of normal; response to slightly higher intensity red flash (0.3 cd.s/m2) shows conserved response of cone-specific part (b1), with similar decrease to two thirds of normal amplitude of rod-specific response (b2); responses to high-intensity flashes (3.0 cd.s/m2 and 10.0 cd.s/m2) show similar reduction to two thirds of normal for both a- and b-waves with only minor delay of responses. Photopic ERG: response to a transient high-intensity flash (3.0 cd.s/m2) shows mild delay of b-wave (right column top trace) and mild delay to a 30 Hz high intensity (3.0 cd.s/m2) flicker stimulus (right column bottom trace). (Bottom traces) Normal reference traces of a healthy control subject.

The elder son was first examined at the age of 21. He had nyctalopia at the age of six years. His general medical history was unremarkable. Best-corrected visual acuity (BCVA) was 20/20 in both eyes. Slit-lamp examination was unremarkable. Fundoscopy showed attenuated retinal blood vessels, peripheral outer retinal atrophy and RPE alterations together with spicular intraretinal pigment migrations. OCT imaging confirmed perimacular outer retinal atrophy and demonstrated bilateral cystoid macular edema^14^. On blue-light autofluorescence imaging (BAF) a mottled aspect of a mostly hypo-autofluorescent retinal periphery, with a small hyperautofluorescent ring surrounding the central macula was seen (Fig. 2). Near-infrared autofluorescence imaging showed a fairly homogeneous hyperautofluorescent central macular area with diffuse hypo-autofluorescence around it. Both eyes were affected equally on fundoscopy and autofluorescence imaging (Fig. 2). Goldmann visual fields showed a normal central sensitivity and a loss of sensitivity in both the pericentral and midperipheral regions, however with normal peripheral limits. Color vision testing was normal. ISCEV-standard full-field flash ERG showed absent scotopic rod-specific responses, significantly decreased combined rod-cone responses to intense flashes and significantly decreased and delayed photopic cone-specific responses (Fig. 3). A clinical diagnosis of RP was made based on all of the findings above.

#### Individual III.2

The younger son was first seen at the age of 19. He complained of nyctalopia and photophobia since he was a toddler. His general medical history revealed an attention deficit disorder (ADD). His BCVA was 20/20 in both eyes. Slit-lamp examination was unremarkable. Fundoscopy showed spicular intraretinal pigment migration and RPE alterations in the retinal periphery. Autofluorescence imaging with blue light revealed a mottled hypo-autofluorescent retinal periphery. Around the macula, a small, hyperautofluorescent ring with a hypo-autofluorescent zone around it was observed. Goldmann visual fields showed decreased pericentral sensitivity with normal peripheral limits. Color vision testing was normal. ISCEV-standard full-field flash ERG revealed considerably reduced and delayed scotopic rod-specific and photopic cone-specific all responses were decreased and delayed (data not shown). A diagnosis of RP was made based on the above findings. Imaging and functional testing showed individual III.2 was less severely affected than his elder brother (III.1). Both eyes were affected equally.

#### Sisters III.3 and III.4

Both had a completely normal extensive ophthalmological examination. The *RHO* c.404G > C p.(Arg135Pro) variant was not found in both individuals.

#### Individual II.1

Individual II.1, the mother of the proband was first examined thoroughly at the age of 45. She had undergone radial keratotomy to correct moderate myopia in both eyes in her twenties. BCVA was 20/20 in both eyes. Slit-lamp biomicroscopy showed scars of the radial keratotomies in both eyes, but was otherwise unremarkable. Only when questioned specifically about symptoms of RP, she reported difficulty navigating at night, as well as mild photophobia. Fundoscopy showed bilateral, but only very limited sectors of intraretinal pigment migration and RPE alterations, especially nasally to the optic disc. Blue light autofluorescence imaging revealed patches of hyperautofluorescence randomly distributed and interspersed by normal areas throughout the retina in both eyes. The left eye was more affected than the right eye. (Fig. 2). This patchy distribution is a typical finding seen in female carriers of X-linked RP, due to the female X-inactivation, also known as lyonization^15,16^. Goldmann visual fields were normal except for a very mild decrease in sensitivity in the midperipheral fields. ISCEV-standard full field-flash ERG revealed a reduction of the amplitudes of scotopic, rod-specific and mixed rod-cone responses as well as photopic cone-specific responses to 70% of normal values, with a mild delay of the peak times for all (Fig. 3). Based on the findings in II.1 and her sons, and on phenotypes in this family, as well as the pedigree, an X-linked RP was suspected.

### Genomic profiling

**Figure 4:**
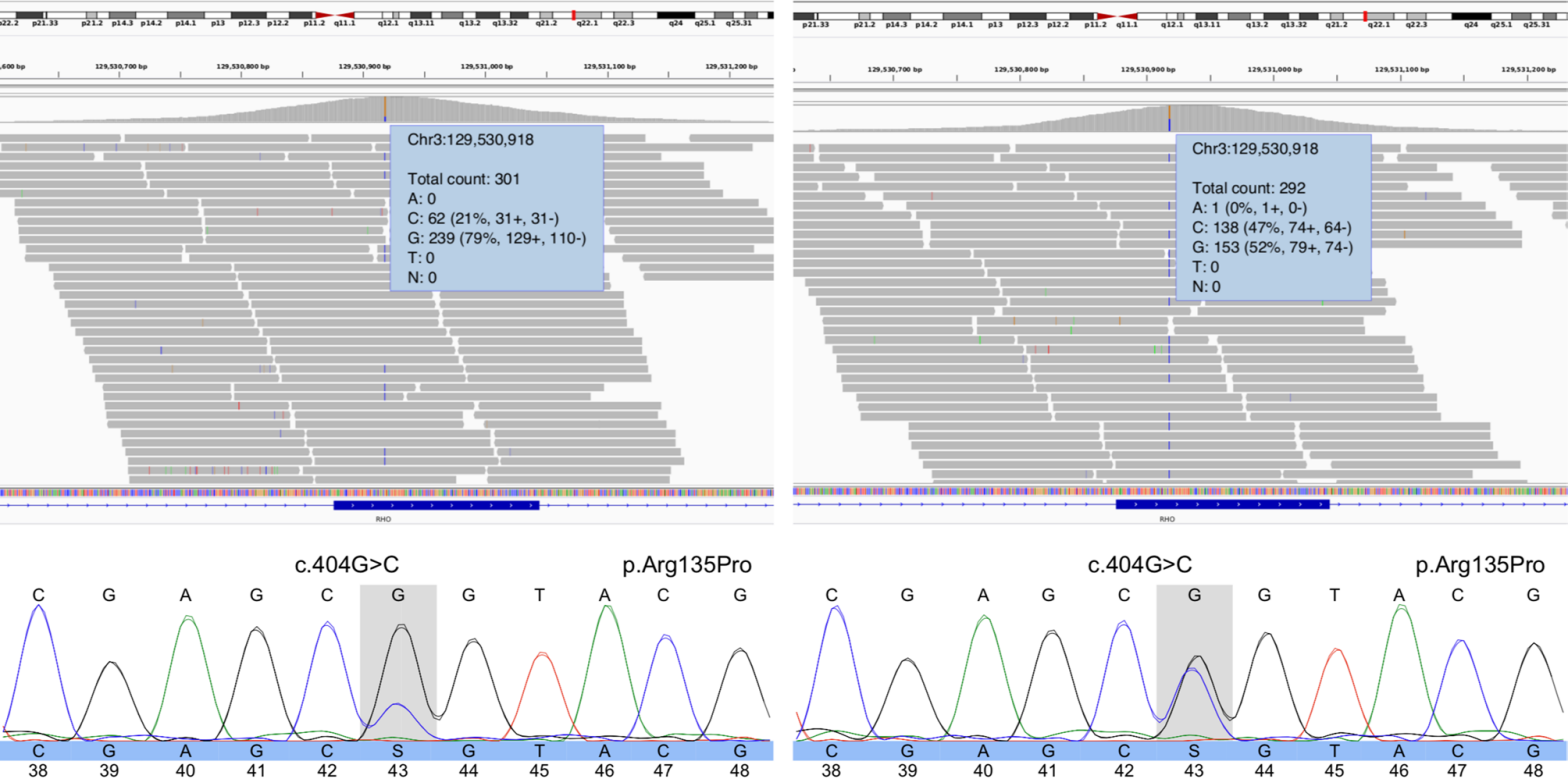
Genomic testing. (Left) Whole exome sequencing reads visualized in the Integrative Genomics Viewer (IGV), showing the *RHO* variant c.404G > C p.(Arg135Pro) in a mosaic state (variant allele frequency [VAF] of ~ 21%) in the mother (II.1) (top). Sanger sequencing confirmation of the mosaic variant is shown at the bottom. (Right) IGV view of the *RHO* variant c.404G > C p.(Arg135Pro) in a heterozygous state (VAF: ~ 47%) in the son (III.1) (top). Sanger sequencing confirmation of the heterozygous variant is shown at the bottom (III.1).

**Figure 5:**
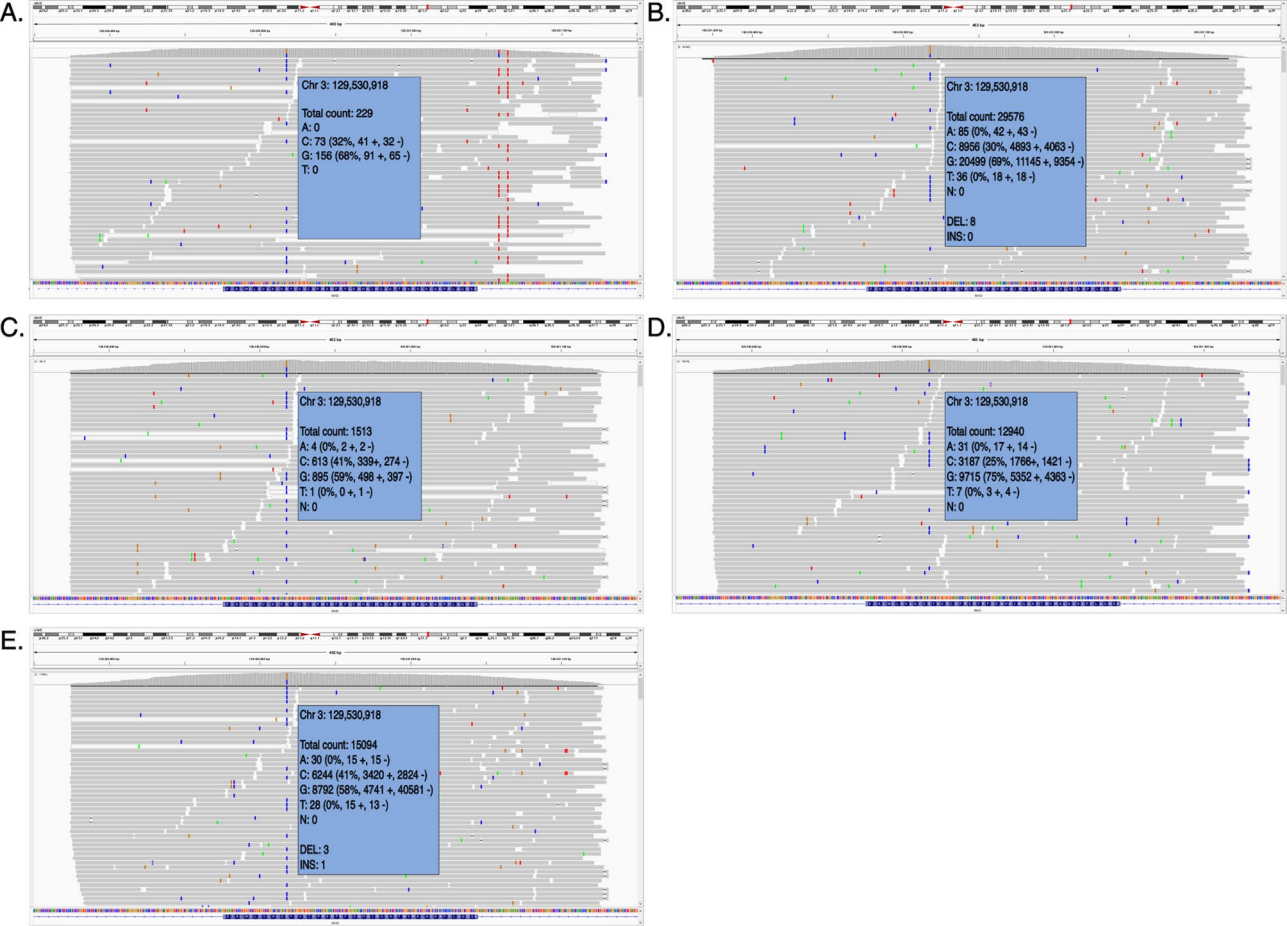
Mosaicism in multiple tissue types. Massively parallel sequencing reads visualized in the Integrative Genomics Viewer (IGV), showing the *RHO* variant c.404G > C p.(Arg135Pro) in a mosaic state in multiple tissues. (**A**) blood sample 1: variant allele frequency (VAF) of 31,97,279%. (**B**) blood sample 2: VAF of 30,4457%. (**C**) buccal mucosa sample 1: VAF of 40,9042%. (**D**) buccal mucosa sample 2: VAF of 25,0641%. (**E**) Sample of hair follicle: VAF of 41, 72,123%.

Given the suspicion of XLRP, genetic testing for XLRP was requested for individual III.1. Panel testing for *RPGR* (including ORF15)*, RP2,* and a deep intronic *OFD1* mutation did not reveal any (likely) pathogenic variants leading to further genetic testing. RetNet-WES analysis in both III.1 and his mother II.1 revealed a known variant in the *RHO* gene, transversion c.404G > C, leading to a missense change p.(Arg135Pro). Interestingly, the *RHO* variant was shown to occur in a mosaic state (variant allele frequency (VAF) of ~ 21%) in the mother (II.1) (Fig. 4). This was confirmed by Sanger sequencing. To investigate the mutation load in other tissues than blood in an accurate way, targeted NGS, characterized by a high coverage, was performed on DNA from maternal leucocytes, buccal cells and hair follicles (II.1) and revealed a VAF ranging from 25.06 to 41.72% in the different tissues, with the highest VAF found in hair follicles and the lowest in the patient’s blood (Fig. 5).

The son was shown to be heterozygous for the *RHO* variant (Fig. 4). Targeted testing showed presence of the variant in both affected brothers (III.1 and III.2) and absence in the unaffected sisters (III.3 and III.4). This variant is a known *RHO* missense variant, already previously reported in ADRP^2,15^. Classification of the variant following ACMG and ACGS criteria revealed this is a likely pathogenic variant (class 4) (Table 1)^9–12^.

**Table 1:** Variant classification using ACMG 2015 and ACGS guidelines.

Gene	*RHO*
RefSeq transcript ID	NM_000539.3
g. notation (HGVS) (GRCh38/hg38)	g.129530918G > C
c. notation (HGVS)	c.404G > C
p. notation (HGVS)	p.(Arg135Pro)
Criteria	> = 3 moderate pathogenic arguments (PM1, PM2, PM5)
Population data	PM2: The variant is NOT present in gnomAD
Genotype and phenotype of the patient	PP4_PP: the phenotype of the patient & family history is specific to one monogenic disorder caused by this gene
Literature and databases	PP5: variant is pathogenic following reliable sources ClinVar ID: 18,175,313, variant described in patients with ADRP
Computational predictions	PP3: prediction programs suggest a deleterious effect of the missense variant PM5: missense variant in a codon in which another pathogenic variant has been found (known pathogenic variant: p.(Arg137Ala) and variant p.(Arg137Cys). Pathogenic missense variant: c.404G > T p.(Arg135Leu) [transcript NM_000539.3]
Functional data	PM1: missense variant is located in mutation hotspot and/or domain in which no benign variants have been reported. * Hot spot/type domain: 7_tm1
Segregation data	PP1: the variant co-segregates with disease in several (additional) affected family members (1–2 family members). Number of affected family members with the variant: 3 (2 additional). Number of unaffected family members without the variant
Allelic data	Not applicable
Conclusion	CLASS 4 (Likely Pathogenic)

PM, pathogenic moderate; PP, pathogenic supporting; PS, pathogenic strong.

## Discussion

We report a family including a female with a suspected X-linked RP carrier-like phenotype, who proved to be mosaic for the dominant variant c.404G > C p.(Arg135Pro) in the *RHO* gene, and her two sons with a classic RP phenotype, heterozygous for the same *RHO* variant.

RP can be inherited in an autosomal dominant, autosomal recessive, or X-linked fashion, depending on the gene involved. Analysis of the pedigree can be performed as a first step to discriminate between the different modes of inheritance. In ADRP, vertical transmission can be seen. In such families, the patients often display a similar disease course and comparable clinical manifestations. However, specific cases of ADRP with variable expressivity and non-penetrance have been described. For example, in *PRPF31*-associated ADRP, different patients from the same family with the same mutation can have a variable disease severity or can be even totally asymptomatic^17^. In XLRP, affected male individuals usually display a more severe and early-onset phenotype, while most female carriers show either RP characteristics later in life or stay asymptomatic^15^. In the latter case, thorough functional testing with electroretinography, BAF and fundus imaging, will reveal retinal abnormalities.

In the family reported here, BAF imaging in the mother revealed patches of hyper-autofluorescence randomly distributed and interspersed by normal areas throughout the retina in both eyes. This is a typical finding in female carriers of XLRP, due to X-inactivation^18^. Interocular asymmetry in the female proband was also a clinical aspect suggestive of XLRP. Indeed, 9% of 125 females heterozygous for an *RPGR* mutation show interocular asymmetry^6^. Given this clinical presentation and the RP phenotype seen in her sons, genetic testing of the XLRP genes was conducted. However, no mutation was found. Subsequent RetNet-WES analysis in III.1 and his mother II.1 identified a variant in the *RHO* gene c.404G > C p.(Arg135Pro). Interestingly, mosaicism of this variant was shown in the mother II.1, estimated to be 21% on DNA from leukocytes, and heterozygosity was shown in her affected son III.1. Co-segregation of the variant with the disease was shown.

Genetic mosaicism is characterized by two or more sets of cell lines with different genotypes in the same individual, resulting from a postzygotic mutation. A variety of factors, including the biological function of the gene, the intrinsic effect of the mutation, the moment when the mutation occurred, and its tissue distribution will determine the type of mosaicism and its phenotypic consequences. This was demonstrated by Cao et al*.* who investigated the allelic fraction and clinical effects of 120 clinically relevant mosaic single nucleotide variants. They showed that an alternate allele fraction (AAF) of 13–24% for mosaic variants in *MTOR* and *PIK3CA* could lead to Smith-Kingsmore syndrome, Cowden syndrome 5, and/or megalocephaly-capillary malformation-polymicrogyria syndrome, while a comparable AAF of 16–30% for mosaic variants in *CACNA1A* was found in asymptomatic parents of children affected with epileptic encephalopathies^19,20^.

Mosaicism can lead to a wide spectrum of phenotypes, ranging from pigment variations to neurofibromatosis type I and osteogenesis imperfecta type II^21^. Additionally, a few cases of mosaicism in eye-related phenotypes have been reported. In particular, it has been shown that approximately 15% of sporadic retinoblastoma cases are caused by postzygotic *RB1* mutations, that intrafamilial phenotypic variability in aniridia cases can be explained by *PAX6* mosaicism^22,23^. In 2016, the contribution of *RHO* mutations to ADRP in the Israeli and Palestinian populations was studied. Segregation analysis of a specific RP-causing *RHO* mutation [c.548_638dup p.(Ile214Alafs*147)] revealed that the mutation originated from a mosaic individual who did not show any clinical signs of RP. The degree of mosaicism in this individual was 13%^24^. In contrast, the mosaic individual (II.1) studied here, showed a variable mutation load ranging from 21 to 42% in the different tissues tested (Fig. 5). Indeed, it has been reported that some pathogenic mosaic variants differ in their abundance depending on the tissue, which may be explained partly by differential negative and positive selective pressures in different tissues^21,25,26^. Moreover, a more accurate quantification of mosaicism, even at a lower degree, is possible using deep NGS^27^. Individual II.1 had phenotypical signs of sectorial RP and a clearly less severe phenotype than that observed in her affected sons. It therefore seems that when a certain threshold of mutation load in the retina is met, clinical disease becomes manifest. However, it is not possible to know the exact mutation load in the retina and how this would compare with the mutation load in blood cells or other types of cells. While the mutation reported by Beryozkin et al*.* has a predicted loss-of-function effect, with a mutation load expected to be proportional to the amount of gene activity, the mutation identified here is known to have a dominant-negative effect, likely resulting in lost gene activity that is presumably higher than the mutation load^11,15,23^.

When counselling mosaicism, the recurrence risk can theoretically be between 0 and 50%, depending on the mutation load in the gametes. In case of somatic mosaicism, the mutation cannot be transmitted to the next generation. In case of germline mosaicism however, a clinically unaffected person can transmit the mutation to an offspring. A combination of both somatic and gonadal mosaicism is also possible. Pre-implantation genetic testing should therefore be discussed as a reproductive option.

In conclusion, we report two male siblings with typical signs of RP and their mother with clinical characteristics highly suggestive for XLRP. While XLRP testing was negative, WES revealed mosaicism of *RHO* variant c.404G > C p.(Arg135Pro) in the mother and heterozygosity of this variant in her affected sons. This study emphasizes that mosaicism for a dominant mutation should be considered in families suggestive of XLRP but testing negative.
